# Neurocutaneous melanosis

**DOI:** 10.1590/0100-3984.2015.0128

**Published:** 2016

**Authors:** Bruno Lima Moreira, Thiago Grunewald, Auro Augusto Junqueira Côrtes, Victor Hugo Rocha Marussi, Lázaro Luís Faria do Amaral

**Affiliations:** 1 Hospital Beneficência Portuguesa de São Paulo, São Paulo, SP, Brazil.

Dear Editor,

A 12-year-old male patient presented with a delay in neuropsychomotor development that had been diagnosed in the first year of life. Two years prior (at 10 years of age), he had undergone ventricular shunt placement because of hydrocephalus. Six months prior to the visit reported here, he had experienced episodes of seizures. Physical examination revealed multiple cutaneous nevi (Figure 1A). Cerebrospinal fluid examination showed an elevated level of protein (1359.7 mg/dL; reference range: 15.0-45.0 mg/dL) and revealed the presence of epithelioid cells. Magnetic resonance imaging (MRI) of the brain (Figures 1B, 1C, and 1D) showed extensive, bilateral, asymmetric leptomeningeal thickening, mainly in the cortical sulci of the right cerebral hemisphere, with spontaneous T1 hyperintense signal (presumably melanin) and diffuse enhancement after intravenous administration of gadolinium contrast. The MRI scan also showed involvement of the brain parenchyma, characterized by spontaneous T1 hyperintense signal in the amygdaloid nuclei and in the cortex, probably due to melanin/melanocyte deposits. Although meningeal biopsy was not performed, a presumptive diagnosis of neurocutaneous melanosis (NCM) was considered.

**Figure 1:**
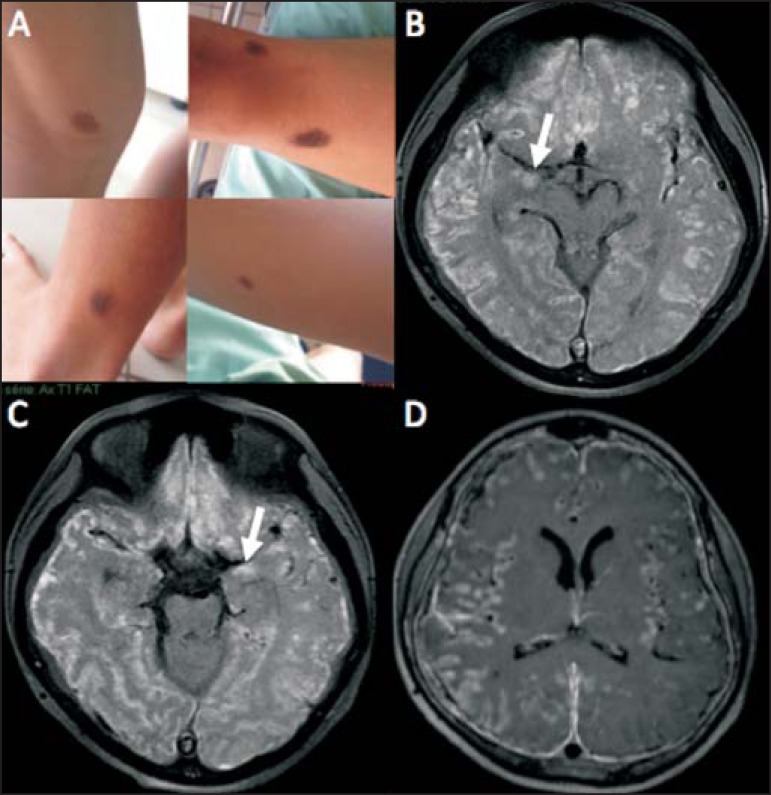
**A:** Multiple cutaneous nevi seen on physical examination. **B,C:** Gadolinium-enhanced axial T1-weighted MRI sequence with fat suppression showing hyperintense signals along the cortical sulci of the cerebral hemispheres, presumably due to diffuse leptomeningeal lesion with melanin content, together with areas of high signal intensity in the amygdaloid nuclei (arrows) and in the cerebral cortex, which likely correspond to parenchymal involvement by melanin/melanocyte deposits in the context of NCM. **D:** Gadolinium-enhanced axial T1-weighted MRI sequence with fat suppression showing diffuse enhancement of the leptomeninges along the cortical sulci of both cerebral hemispheres, especially on the right side.

NCM is a rare sporadic neuroectodermal syndrome, first described by Rokitansky in 1861, and characterized by congenital cutaneous nevi (one large nevus or multiple nevi) associated with benign or malignant central nervous system (CNS) proliferation of melanocytes^(1-4)^. The diagnostic criteria, which were first described by Fox and later revised by Kadonaga and Frieden^(1)^ in 1991, include the combination of all of the following: - a single giant congenital nevus (measuring at its greatest diameter ≥ 20 cm in adults, or ≥ 9 cm on the head or ≥ 6 cm on the trunk in neonates and infants) or multiple (three or more) congenital nevi, accompanied by meningeal melanosis or CNS melanoma; - the absence of cutaneous melanoma, except in patients with a meningeal lesion histologically proven to be benign; - the absence of meningeal melanoma, except in patients with cutaneous lesions histologically proven to be benign.

Approximately 60% to 70% of all individuals with NCM develop symptoms, which usually appear before five years of age^(2)^. Clinically, patients can experience seizures, hydrocephalus, developmental delays, psychiatric disorders, cranial nerve palsies, intracranial hemorrhage, and myelopathy^(1,2,5-7)^. Seizures are the most common initial neurological manifestation^(2)^. Involvement of the CNS can include parenchymal or leptomeningeal lesions, such as melanosis (aggregation of benign melanocytic cells) or melanomas^(5)^.

In NCM, the MRI findings can include hyperintense areas in the temporal lobes on T1-weighted images, diffuse leptomeningeal enhancement of the brain and spine, and mass of malignant melanoma^(4)^. Parenchymal melanosis typically occurs in the temporal lobes (amygdaloid nuclei), cerebellum, or pons; and its lesions usually exhibit a high signal intensity on T1-weighted images and do not commonly show enhancement after contrast administration^(5,7,8)^. The leptomeningeal lesions usually present intermediate to high signal intensity on T1-weighted images, low to intermediate signal intensity on T2-weighted images, high signal intensity on fluid-attenuated inversion recovery (FLAIR) sequence and diffuse enhancement after gadolinum administration. Mass effect, edema, hemorrhage, and necrosis favor the possibility of melanoma and make benign melanocytic lesion less likely^(5)^. Abnormalities in the spine, especially cystic malformations (mainly arachnoid cysts), are relatively common in patients with NCM^(2)^.

The differential diagnoses that can be based on MRI images of the brain include subarachnoid hemorrhage, meningitis, leptomeningeal carcinomatosis, other melanin-containing lesions, and non-melanocytic hemorrhagic tumors. The clinical context and the imaging characteristics will aid in making that differentiation^(4,9)^.

Regardless of the treatment instituted, the prognosis is usually poor, especially in cases with diffuse leptomeningeal involvement^(2,5,6)^.

## References

[1] Kadonaga JN, Frieden IJ (1991). Neurocutaneous melanosis: definition and review of the
literature. J Am Acad Dermatol.

[2] Ramaswamy V, Delaney H, Haque S (2012). Spectrum of central nervous system abnormalities in
neurocutaneous melanocytosis. Dev Med Child Neurol.

[3] Scattolin MA, Lin J, Peruchi MM (2011). Neurocutaneous melanosis: follow-up and literature
review. J Neuroradiol.

[4] Hayashi M, Maeda M, Maji T (2004). Diffuse leptomeningeal hyperintensity on fluid-attenuated
inversion recovery MR images in neurocutaneous melanosis. AJNR Am J Neuroradiol.

[5] Oliveira RS, Carvalho AP, Noro F (2013). Neurocutaneous melanosis. Arq Neuropsiquiatr.

[6] Sabat SB (2010). Teaching NeuroImages: neurocutaneous melanosis. Neurology.

[7] Demirci A, Kawamura Y, Sze G (1995). MR of parenchymal neurocutaneous melanosis. AJNR Am J Neuroradiol.

[8] Fu YJ, Morota N, Nakagawa A (2010). Neurocutaneous melanosis: surgical pathological features of an
apparently hamartomatous lesion in the amygdala. J Neurosurg Pediatr.

[9] Pont MS, Elster AD (1992). Lesions of skin and brain: modern imaging of the neurocutaneous
syndromes. AJR Am J Roentgenol.

